# Identification of a Novel Epithelial–Mesenchymal Transition Gene Signature Predicting Survival in Patients With HNSCC

**DOI:** 10.3389/pore.2021.585192

**Published:** 2021-03-29

**Authors:** Wei Xin, Chaoran Zhao, Longyang Jiang, Dongmei Pei, Lin Zhao, Chengpu Zhang

**Affiliations:** ^1^ Department of Pharmacology, School of Pharmacy, China Medical University, Shenyang, China; ^2^ Liaoning Key Laboratory of Molecular Targeted Anti-tumor Drug Development and Evaluation China Medical University, Shenyang, China; ^3^ Department of Family Medicine, Shengjing Hospital, China Medical University, Shenyang, China

**Keywords:** epithelial-mesenchymal transition, HNSCC, mRNAs, Prognostic, survival

## Abstract

Head and neck squamous cell cancer (HNSCC) is one of the most common types of cancer worldwide. There have been many reports suggesting that biomarkers explored via database mining plays a critical role in predicting HNSCC prognosis. However, a single biomarker for prognostic analysis is not adequate. Additionally, there is growing evidence indicating that gene signature could be a better choice for HNSCC prognosis. We performed a comprehensive analysis of mRNA expression profiles using clinical information of HNSCC patients from The *Cancer* Genome Atlas (TCGA). Gene Set Enrichment Analysis (GSEA) was performed, and we found that a set of genes involved in epithelial mesenchymal transition (EMT) contributed to HNSCC. Cox proportional regression model was used to identify a four-gene (*WIPF1, PPIB, BASP1, PLOD2*) signature that were significantly associated with overall survival (OS), and all the four genes were significantly upregulated in tumor tissues. We successfully classified the patients with HNSCC into high-risk and low-risk groups, where in high-risk indicated poorer patient prognosis, indicating that this gene signature might be a novel potential biomarker for the prognosis of HNSCC. The prognostic ability of the gene signature was further validated in an independent cohort from the Gene Expression Omnibus (GEO) database. In conclusion, we identified a four-EMT-based gene signature which provides the potentiality to serve as novel independent biomarkers for predicting survival in HNSCC patients, as well as a new possibility for individualized treatment of HNSCC.

## Introduction

Head and neck cancer is the sixth most common types of cancer, with about half a million new cases annually diagnosed worldwide [1], among which 350,000 individuals die of it [2]. Although local control rate and quality of life have improved for head and neck squamous cell cancer (HNSCC) owing to advances in surgical techniques and comprehensive treatment techniques, overall survival (OS) has not increased significantly in recent decades. Moreover, the 5-years survival rate of patients with this disease is only 40–50% [3]. In recent years, age, clinical stage, and smoking status which are characteristics emerging as important contributors to the clinical outcome might help improve survival prediction for patients afflicted by HNSCC [4–6]. However, due to the complex molecular mechanisms underlying cancer regulation, the conventional clinical information has limited predictive ability.

Increasing evidence has revealed the important clinical significance of mRNA expression in various pathological and physiological processes of multiple tumor histotypes, including HNSCC. For instance, decreased calpain six expression has been known to be associated with tumorigenesis and poor prognosis of HNSCC [7]. Furthermore, FcGBP expression was upregulated by HPV infection and correlated with longer survival time of HNSCC patients [8]. However, compared with single-gene biomarkers, tumor signatures including several genes have been identified, which might be better choices to facilitate clinical application, provide insights into cancer progression, as well as reveal potentially new therapeutic targets [9, 10]. Thus, it is necessary to establish an expression-based gene signature for predicting survival of HNSCC patients for effective clinical decisions making with respect to optimal treatment regimen.

Metastasis is a complex, highly inefficient, but deadly process that has been under intense investigation in hopes to eliminate distant spread of metastasis and reduce cancer-associated mortality. A key event in promoting stationary tumor cells to migrate and invade is the epithelial mesenchymal transition (EMT) [11]. Specific tumor cell populations with EMT are associated with more aggressive tumor phenotypes and worse outcomes [12, 13]. Therefore, understanding the relationship between EMT and tumor is crucial for elucidating the underlying mechanism of tumorigenesis. Gene Set Enrichment Analysis (GSEA) is not concerned with a limited number of different genes that have significantly changed, but with whether the expression of these detected genes exhibit a common expression pattern in the defined functional groups, and interprets biological information to elaborate its biological significance from another perspective. In the present study, we tried to identify the relationship between the metastatic cascade system and HNSCC by GSEA analysis [14]. As expected, EMT-related risk signature could independently identify patients afflicted by HNSCC with high-risk and poor prognosis.

## Materials and Methods

### Data Acquisition

We downloaded the mRNA expression profiles and clinical information of head and neck squamous cell cancer patients from the TCGA database (https://cancergenome.nih.gov/). A total of 546 samples involving tumor tissue samples and adjacent noncancerous tissues participated in the study, and their corresponding clinical data including age, sex, TNM classification, OS status, as well as disease-free survival (DFS) status were also examined. The general clinical features are listed in Table 1. As an external validation cohort, the independent data set GSE27020 based upon the GPL96 platform and survival information were extracted from the Gene Expression Omnibus (GEO) database (https://www.ncbi.nlm.nih.gov/geo/).

**TABLE 1:** Summary information of clinical characteristics of HNSCC patients in entire TCGA set (n = 494), TCGA validation set 1 (n = 346), TCGA validation set 2 (n = 148).

Clinical feature	TCGA-HNSCC entire cohort (n = 494), n (%)	Patients in validation set 1 (n = 346), n (%)	Patients in validation set 2 (n = 148), n (%)
Gender			
Male	363 (73.48)	255 (73.70)	108 (72.97)
Female	131 (26.52)	91 (26.30)	40 (27.03)
Age			
≥61	253 (51.21)	182 (52.60)	71 (47.97)
＜61	241 (48.79)	164 (47.40)	77 (52.03)
Clinical T			
T1-T2	173 (29.85)	126 (37.39)	47 (33.10)
T3-T4	306 (63.88)	211 (62.61)	95 (66.90)
Clinical N			
N0	235 (49.68)	174 (52.57)	61 (42.96)
N1- N3	238 (50.32)	157 (47.43)	81 (57.04)
Clinical stage			
I-II	111 (23.13)	80 (23.67)	31 (21.83)
III-IV	369 (76.88)	258 (76.33)	111 (78.17)
Grade			
I-II	356 (74.95)	252 (76.12)	104 (72.22)
III-IV	119 (25.05)	79 (23.87)	40 (27.78)
HPV P16 status			
Negative	71 (70.30)	46 (70.77)	25 (69.44)
Positive	30 (29.70)	19 (29.23)	11 (30.56)
Person neoplasm cancer status			
Tumor free	311 (69.73)	212 (68.61)	99 (72.26)
With tumor	135 (30.27)	97 (31.39)	38 (27.74)
New tumor event after initial treatment			
Yes	46 (24.86)	36 (26.67)	10 (20.00)
No	139 (75.14)	99 (73.33)	40 (80.00)
Alcohol history			
Yes	327 (67.70)	229 (67.95)	98 (67.12)
No	156 (32.30)	108 (32.05)	48 (32.88)
Tobacco smoking history			
I-II	277 (57.23)	186 (55.03)	91 (62.33)
III-IV	207 (42.77)	152 (44.97)	55 (37.67)
Lymph node neck dissection			
Yes	402 (81.71)	280 (81.40)	122 (82.43)
No	90 (18.29)	64 (18.60)	26 (17.57)
Lymph vascular invasion			
Yes	119 (35.63)	81 (33.61)	38 (40.86)
No	215 (64.37)	160 (66.39)	55 (59.14)
Perineural invasion			
Yes	162 (46.82)	116 (47.54)	46 (45.10)
No	184 (53.18)	128 (52.46)	56 (54.90)
Radiation therapy			
Yes	118 (67.05)	84 (65.63)	34 (70.83)
No	58 (32.95)	44 (34.38)	14 (29.17)

### Gene Set Enrichment Analysis

GSEA (http://www.broadinstitute.org/gsea/index.jsp) was performed to explore whether the gene sets identified between the two groups showed a significant difference [15, 16]. We conducted GSEA to investigate the differences of biological pathways associated with tumorigenesis and progression between adjacent noncancerous tissue and HNSCC samples by selecting the hallmark gene sets as the reference gene set file. Normalized *p* values (*p* < 0.05) were set as threshold to determine which functions for further investigation.

### Gene Ontology Analysis

We used DAVID (the Database for Annotation, Visualization and Integration Discovery v6.8, https://david.ncifcrf.gov/) to analyze the TCGA-HNSCC cohort, to obtain the enriched pathways for differentially expressed genes between four-mRNA-low-risk and high-risk groups [17].

### Statistical Analysis

Using gene expression profiles as the original data, each gene was normalized by log2 transformed values for further analysis. Univariate Cox regression analysis was used to calculate the association between mRNA expression levels and OS rate of patients. mRNAs were considered significant if their *p*-values were less than 0.05. Candidate genes were fitted in a stepwise multivariate Cox proportional regression model to identify predictive models with optimal interpretation and valuable information. We used the R package “survival” to build a risk score model. The formula for the risk score is described below: Risk score = expression of gene 1*β1+ expression of gene 2*β2+…+expression of gene n*βn. The filtered mRNAs were classified into risky [hazard ratio (HR) >1] and protective (0 < HR < 1) types. All patients were classified into a high-risk and a low-risk group according to the median risk score. Additionally, the correlations between the risk score and clinical features of HNSCC patients were analyzed by using Chi-square test. Time-dependent receiver-operating characteristic (ROC) curves and area under the ROC curve (AUC) values were calculated to measure prognostic accuracy. Afterward, we applied the R package “caret” to randomly divided TCGA-HNSCC patients into two sets (TCGA validation set 1, *n* = 346 and TCGA validation set 2, *n* = 148) at a ratio of 7:3. The prognostic signature was subsequently validated in both internal validation sets and external independent data set GSE27020. All statistical analyses were performed using SPSS 24.0 and GraphPad Prism8 software.

## Results

### Using GSEA for Preliminary Screening of Genes

**FIGURE 1:**
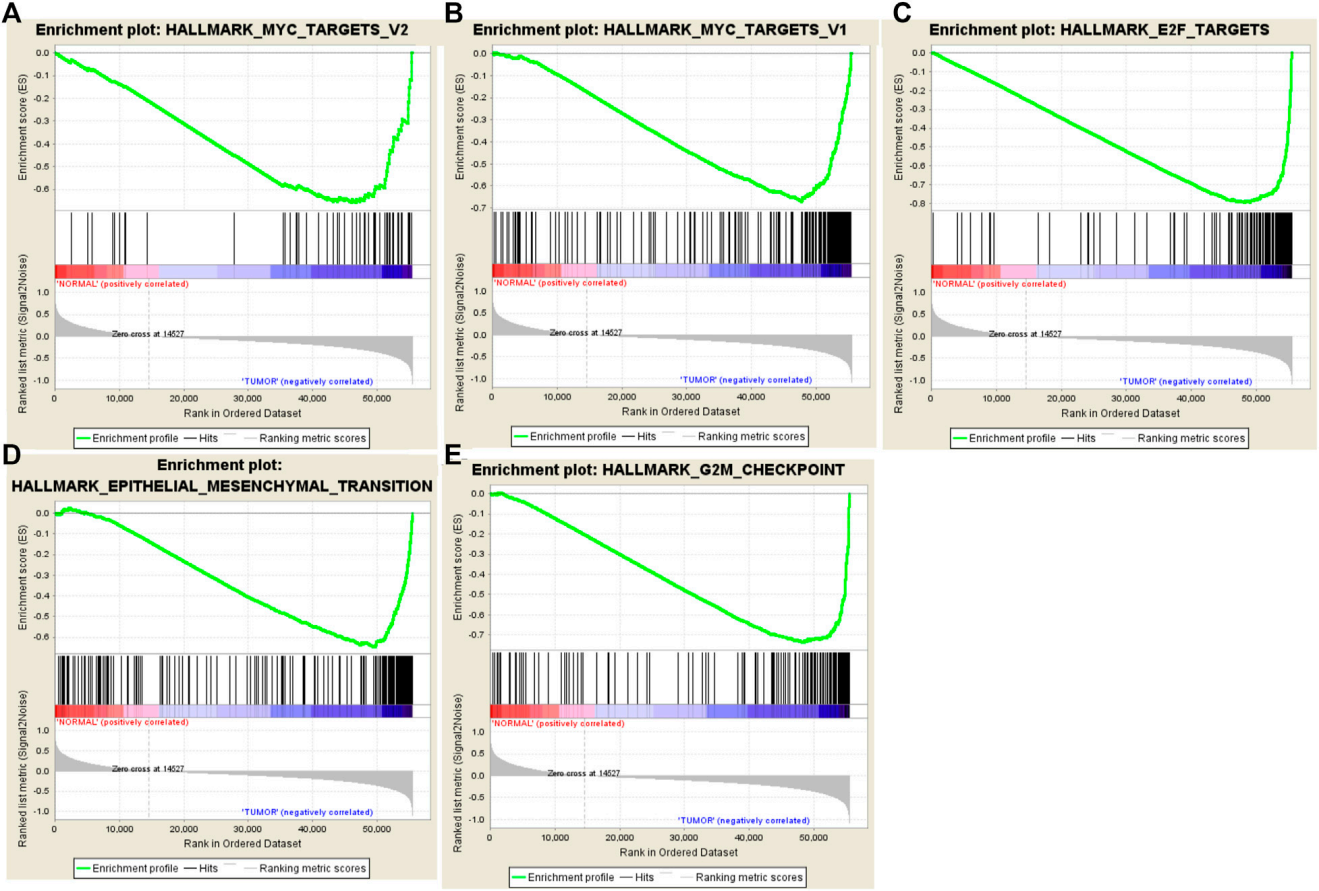
Enrichment plots of five gene sets which were significantly different in normal and HNSCC tissues using GSEA. [Including **(A)** MYC targets V2; **(B)** MYC targets V1; **(C)** E2F targets; **(D)** Epithelial Mesenchymal Transition; **(E)** G2/M checkpoint].

Clinical features of TGCA-HNSCC cohort (*n* = 546), and expression data sets of HNSCC patients were obtained from TCGA. GSEA was applied to explore whether the gene sets identified as EMT, G2/M checkpoint, E2F targets, MYC targets V2 and MYC targets V1 showed statistically significant differences between HNSCC samples and adjacent normal tissues. We found that all the five gene sets were significantly enriched with normalized *p*-values < 0.05 (Figure 1; Table 2). We then selected the EMT gene set which was of our interest (*p* = 0.013), containing 94 core genes (Supplementary Table S1) for further analysis.

**TABLE 2:** Gene sets enriched in HNSCC.

GS follow link to MSigDB	Size	ES	NOM *p*-value	Rank at Max
E2F Targets	197	−0.8	0.002	7,217
G2M Checkpoint	196	−0.74	0.002	7,248
Epithelial mesenchymal transition	197	−0.65	0.013	5,720
MYC Targets V1	197	−0.67	0.016	7,656
MYC Targets V2	58	−0.66	0.041	9,384

### Identification of EMT mRNAs Related to HNSCC Patient Survival

We first performed a preliminary screening of 94 genes by univariate Cox regression analysis and obtained 12 genes (Supplementary Table S2) with a *p*-value <0.05. Next, using multivariate Cox regression analysis, we further examined the association between the 12 mRNA expression profiles and patient survival. Subsequently, we confirmed four-mRNA signature (WIPF1, PPIB, BASP1 and PLOD2), with a *p*-value < 0.001, as an independent prognostic indicator of HNSCC. Filtered mRNAs were classified as risky type (PPIB, BASP1 and PLOD2) with *β*(cox) was >0 and shorter survival, and a protective type (WIPF1), with *β*(cox) was <0 and longer survival (Table 3).

**TABLE 3:** Detailed information of four prognostic mRNAs significantly associated with OS in patients with HNSCC.

mRNA	Ensemble Id	Location	β(cox)
WIPF1	ENST00000359761.7	chr2:174,562,204–174,682,883	−0.23734
PPIB	ENST00000300026.3	chr15:64,155,812–64,163,205	0.19805
BASP1	ENST00000322611.3	chr5:17,217,560–17,276,834	0.09904
PLOD2	ENST00000360060.7	chr3:146,069,444–146,161,167	0.15977

Four-mRNA risk model *p*-value = 1.166e-05.

**FIGURE 2:**
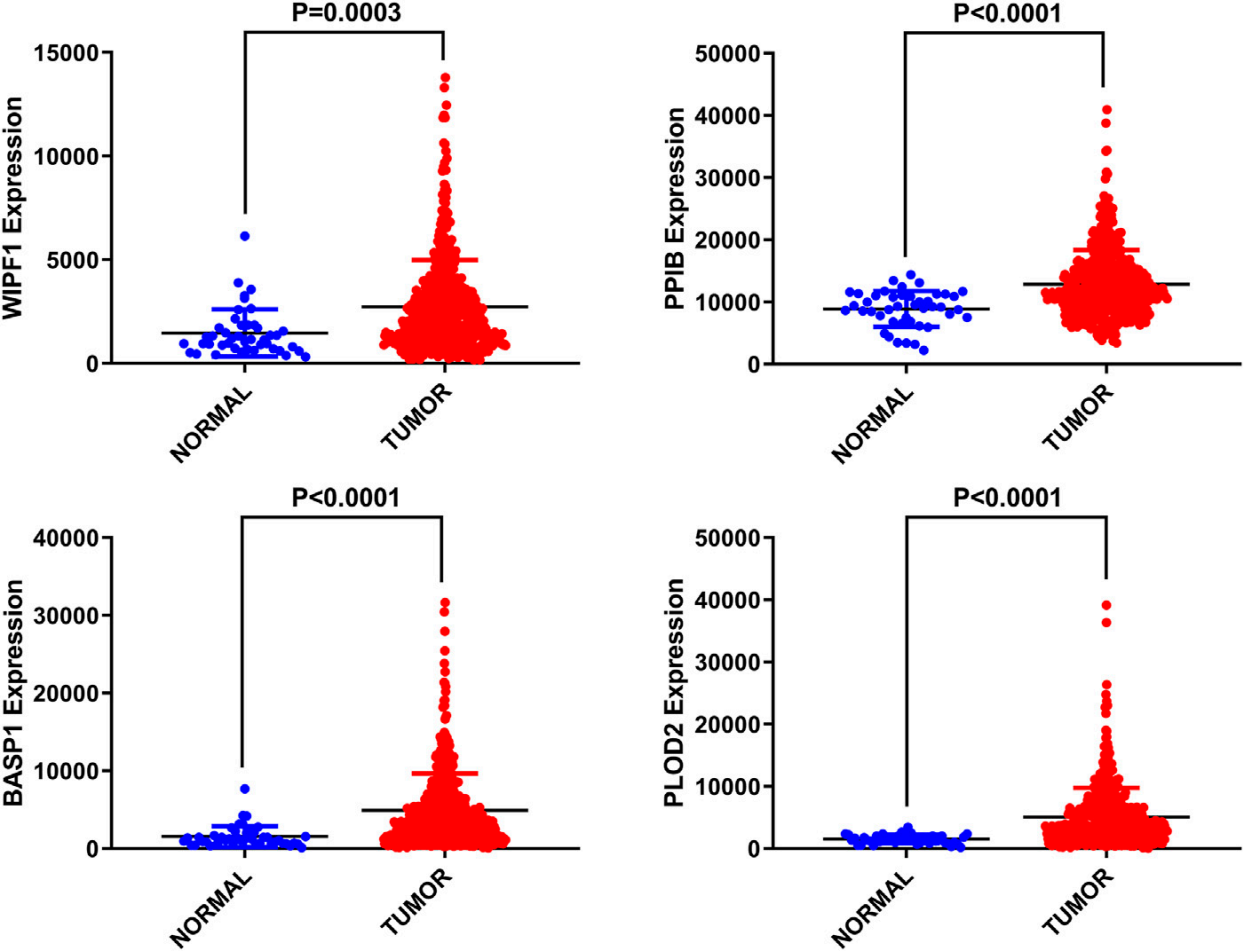
Differential expression of four selected genes.

Differential expression of four genes in adjacent normal tissues was also investigated compared to HNSCC tissues. We found that these four genes were up-regulated in tumor tissues with significant differences (*p* < 0.05, Figure 2).

### Four-mRNA Signature Was Constructed to Predict the Prognosis of Patients

**FIGURE 3:**
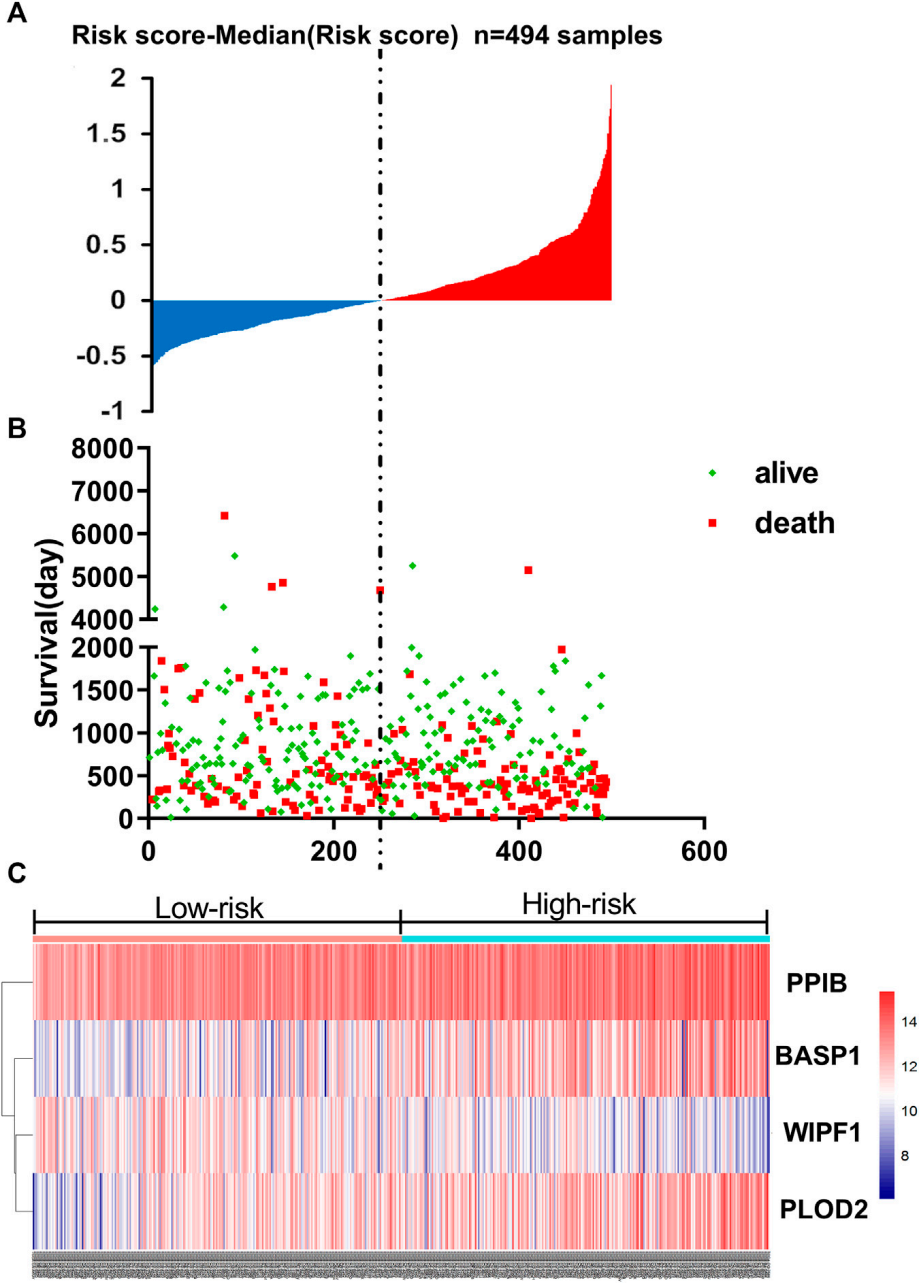
The four-mRNA signature related to risk score predicts OS in patients with HNSCC **(A)** mRNA risk score distribution in each patient. **(B)** Survival (in days) of patients in order of the value of risk scores. **(C)** A heatmap showing the expression profile of the four selected genes.

Based on the linear combinations of the expression level, the formula for prognostic risk rating was established, and the expression level was weighted by the regression coefficients from the multivariable Cox regression analysis. Risk score = 0.19805 * expression of PPIB + 0.09904 * expression of BASP1 + 0.15977 * expression of PLOD2−0.23734 * expression of WIPF1. Every HNSCC patient had only one risk score. We calculated the scores and then ranked the patients in order of increased risk scores. Based on the median point (0.994727), we then classified them into high-risk and low-risk groups (Figure 3A). The distribution and survival status for each patient is shown in Figure 3B. The median survival time of the high-risk and low-risk group was 998 and 1762 days, respectively. In addition, the mortality rate of the high-risk group was 48.6%, whereas the corresponding rate in the low-risk group was 38.7%. The mortality rate of patients with a high-risk score was higher than that of patients with a low-risk score (*p* = 0.0268). According to the univariate Cox regression analysis of OS, compared to the low-risk group, a 1.573-fold increased risk of death (95% CI 1.200–2.062, *p* = 0.001) was determined for the high-risk group. Further, the heat map displayed the expression profiles of four-mRNAs (Figure 3C). HNSCC patients with an increased risk score showed significantly upregulated expression of high-risk type mRNA (PPIB, BASP1 and PLOD2); in contrast, the expression of protective type mRNA (WIPF1) was down-regulated.

### Risk Scores Generated by Four-mRNA Signature as Independent Prognostic Indicators in HNSCC

**FIGURE 4:**
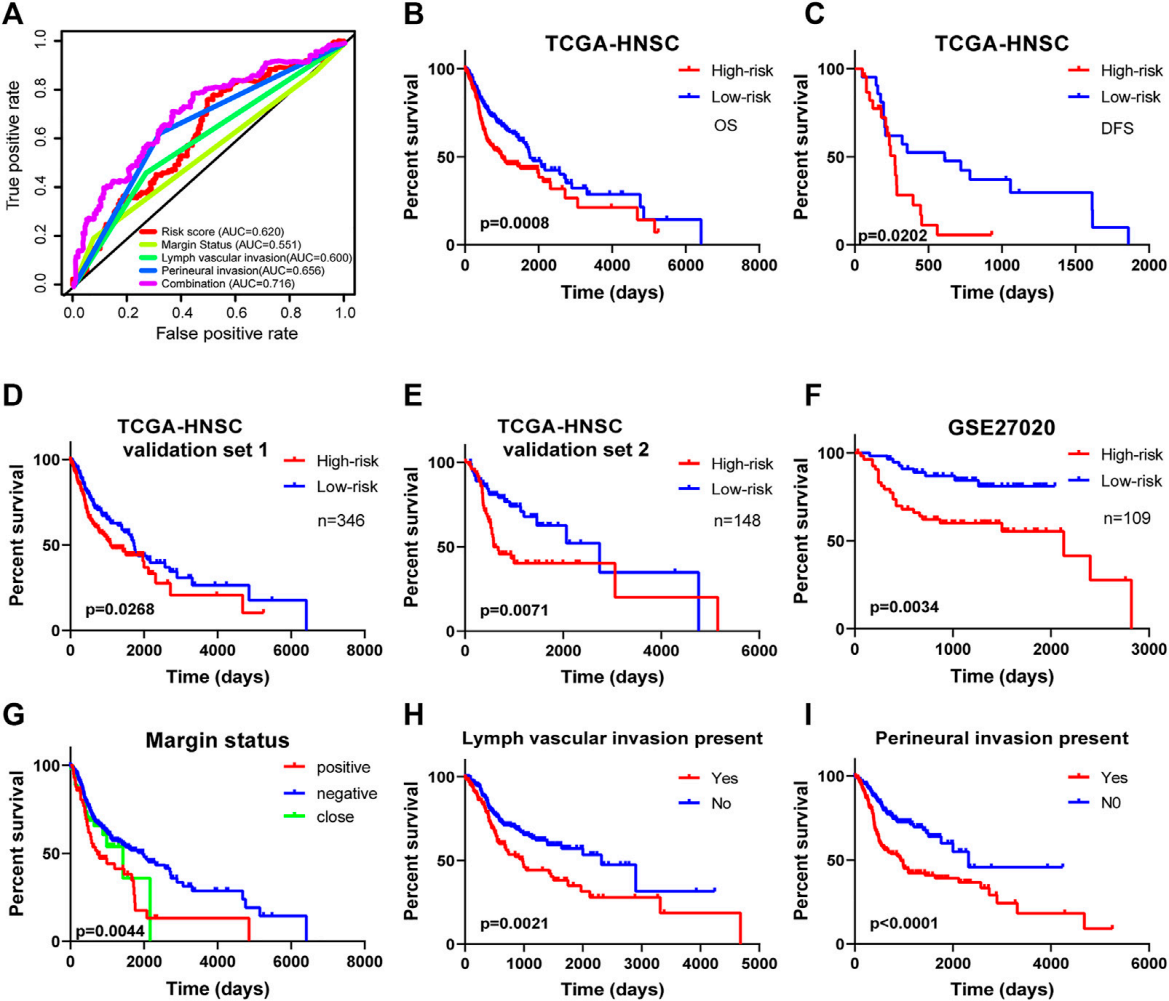
Kaplan-Meier survival analysis for patients with HNSCC stratified by the prognostic signature. **(A)** The receiver operating characteristic (ROC) for EMT-based prognostic signature, margin status, lymph node vascular invasion and perineural invasion only, and the EMT-based risk score combined with the above clinical parameters for OS of HNSCC patients. **(B)** The Kaplan-Meier curve of OS for patients in TCGA-HNSCC cohort divided into high-risk and low-risk groups. **(C)** The Kaplan-Meier curve of DFS for patients in TCGA-HNSCC cohort divided into high-risk and low-risk groups. **(D)** The Kaplan-Meier curve of prognostic signature for HNSCC patients in TCGA validation set 1 (*n* = 346). **(E)** The Kaplan-Meier curve of prognostic signature for HNSCC patients in TCGA validation set 2 (*n* = 148). **(F)** The Kaplan-Meier curve of OS in the high- and low-risk groups stratified by the four-mRNA signature in the GSE27020. Different clinical features including **(G)** margin status, **(H)** lymph vascular invasion, and **(I)** presence of perineural invasion predicts patient survival.

The results of Chi-square analysis showed that patients in the low-risk group had better clinicopathological parameters, including tumor grade (χ^2^ = 10.619, *p* = 0.014), neoplasm cancer status (χ^2^ = 6.948, *p* = 0.008), new tumor event after initial treatment (χ^2^ = 5.535, *p* = 0.018), compared with those in high-risk cohort (Table 4). Comparison of prognostic values of risk scores with clinical pathology parameters using univariate and multivariate analysis (Table 5). Univariate Cox analysis showed that the high-risk group was closely associated with poor survival of HNSCC patients (HR = 2.177, 95% CI 1.627–2.912, *p* < 0.001). After adjusting for clinical and pathological parameters, we found that the risk score (HR = 1.844, 95% CI 1.198–2.836, *p* = 0.005), margin status (HR = 1.410, 95% CI 1.107–1.798, *p* = 0.005), and presence of perineural invasion (HR = 2.218, 95% CI 1.490–3.302, *p* < 0.001) were still independent prognostic indicators in the multivariate Cox analysis. To assess the capabilities of the four-EMT-based classifier to predict OS of HNSCC, we plotted the ROC curves and the AUC value for the long-time survival at 3 years of our signature was 0.620 (Figure 4A). In consideration of the role of classical parameters in clinical practice, we combined the EMT-based model and the classical clinical parameters to predict OS of HNSCC, and the AUC value was 0.716, indicating that this model was more accurate than models enrolled in EMT-related signature or clinical parameters solely.

**TABLE 4:** After grouping demographic and clinical characteristics of TCGA-HNSCC cohort.

Clinical feature	Risk score		χ^2^	*p*
	High risk n (%)	Low risk n (%)		
Gender			0.208	0.649
Male	183 (74.4%)	180 (72.6%)		
Female	63 (25.6%)	68 (27.4%)		
Age			0.453	0.5008
≥61	123 (49.8%)	131 (52.8%)		
＜61	124 (50.2%)	117 (47.2%)		
Clinical T			4.21	0.240
T1	11 (4.6%)	22 (9.0%)		
T2	67 (28.1%)	73 (29.8%)		
T3	66 (27.7%)	64 (26.1%)		
T4-T4b	94 (39.5%)	86 (35.1%)		
Clinical N			1.601	0.206
N0	112 (49.8%)	123 (51.5%)		
N1- N3	133 (50.2%)	116 (48.5%)		
Clinical stage			3.283	0.350
I	7 (2.9%)	12 (5.0%)		
II	46 (19.3%)	46 (19.0%)		
III	47 (19.8%)	55 (22.7%)		
IV	139 (58.0%)	129 (53.3%)		
Grade			10.619	0.014
I	28 (11.7%)	33 (14.0%)		
II	164 (68.3%)	131 (55.7%)		
III	48 (20.0%)	69 (29.4%)		
IV	0 (0%)	2 (0.9%)		
HPV P16 status			2.760	0.097
Negative	34 (79.1%)	37 (63.8%)		
Positive	9 (21.0%)	21 (36.2)		
Person neoplasm cancer status			6.948	0.008
Tumor free	139 (63.8%)	173 (75.2%)		
With tumor	79 (36.2%)	57 (24.8%)		
New tumor event after initial treatment			5.535	0.018
Yes	32 (31.7%)	14 (16.7%)		
No	69 (68.3%)	71 (83.3%)		
Alcohol history			1.206	0.2722
Yes	157 (65.4%)	171 (70.0%)		
No	83 (34.6%)	73 (30.0%)		
Tobacco smoking history			0.8138	0.846
I	58 (24.3%)	52 (21.4%)		
II	79 (33.0%)	87 (35.8%)		
III	34 (14.2%)	37 (15.2%)		
IV	68 (28.5%)	67 (27.6%)		
Lymph node neck dissection			1.461	0.226
Yes	207 (83.8%)	195 (79.6%)		
No	40 (16.2%)	50 (20.4%)		
Lymph vascular invasion			1.141	0.286
Yes	55 (32.7%)	64 (38.3%)		
No	113 (67.3%)	103 (61.7%)		
Perineural invasion			1.559	0.212
Yes	91 (50%)	71 (43.3%)		
No	91 (50%)	93 (56.7%)		
Radiation therapy			0.2363	0.627
Yes	64 (66.7%)	54 (67.5%)		
No	32 (33.3%)	26 (32.5%)		

**TABLE 5:** Univariable and multivariable analyses for each clinical feature.

Univariate analysis				
Clinical feature	HR	95% Cl	*p*-value	HR
Risk score	2.177	1.627–2.912	<0.001	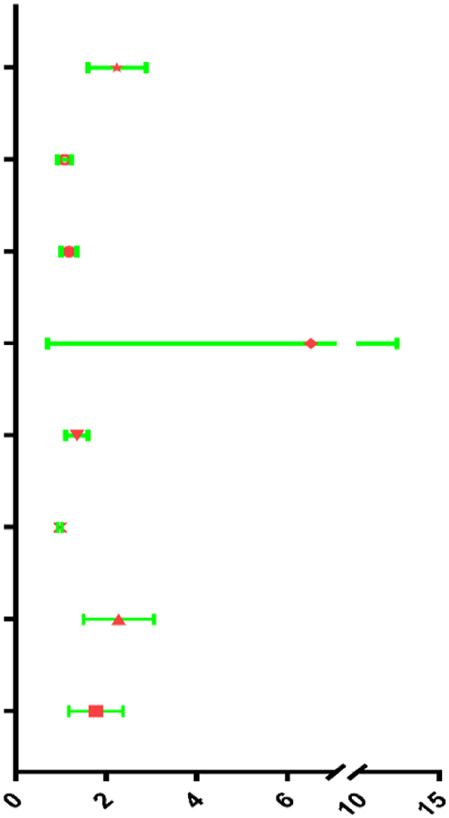
Clinical T	1.078	0.936–1.242	0.296	
Clinical N	1.173	1.012–1.360	0.034	
Clinical M	4.785	1.762–12.994	0.002	
Margin status	1.340	1.123–1.599	0.001	
Tumor subsite	0.980	0.937–1.025	0.980	
Perineural invasion present	2.186	1.545–3.095	<0.001	
Lymph vascular invasion present	1.704	1.211–2.397	0.002	
**Multivariate analysis**				
**Clinical feature**	**HR**	**95% Cl**	***p*-value**	**HR**
Risk score	1.844	1.198–2.836	0.005	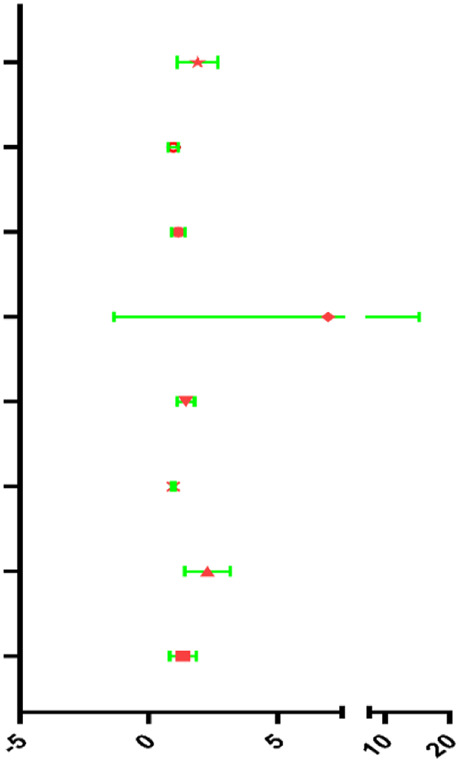
Clinical T	0.926	0.748–1.146	0.479	
Clinical N	1.161	0.932–1.446	0.182	
Clinical M	3.744	0.839–16.713	0.084	
Margin status	1.410	1.107–1.798	0.005	
Tumor subsite	0.971	0.913–1.033	0.347	
Perineural invasion present	2.218	1.490–3.302	<0.001	
Lymph vascular invasion present	1.250	0.839–1.861	0.273	

### Kaplan-Meier Curves for Survival Predicted Four-mRNA Signature

Kaplan-Meier curves and the log-rank method showed that patients with high**-**risk scores had poorer OS time (*p* = 0.0008; Figure 4B). Meanwhile, the signature showed great utility in predicting DFS with *p*-value of 0.0202 (Figure 4C). Additionally, in TCGA validation set 1 (*n* = 346) and validation set 2 (*n* = 148), the Kaplan-Meier curves displayed significant differences between high-risk and low-risk patients (*p* < 0.05, Figures 4D,E). We also extracted HNSCC afflicted patients from the GSE27020 dataset (*n* = 109) and applied the same formula to validate the ability of the four-EMT-based classifier predicting OS of HNSCC. As expected, patients in the low-risk group had a significantly longer survival time compared to the high-risk group (*p* = 0.0034; Figure 4F). Univariate Cox regression analysis of OS showed that several clinicopathological parameters were effective predictors of HNSCC survival, including clinical N, clinical M, margin status, lymphangitic invasion and perineural infiltration. Then Kaplan-Meier curve was used to verify the above conclusion, and the results were self-consistent (Figures 4G–I). According to the curve, patients with lymphangitic invasions and peripheral nerve invasions had a poor prognosis during follow-up.

**FIGURE 5:**
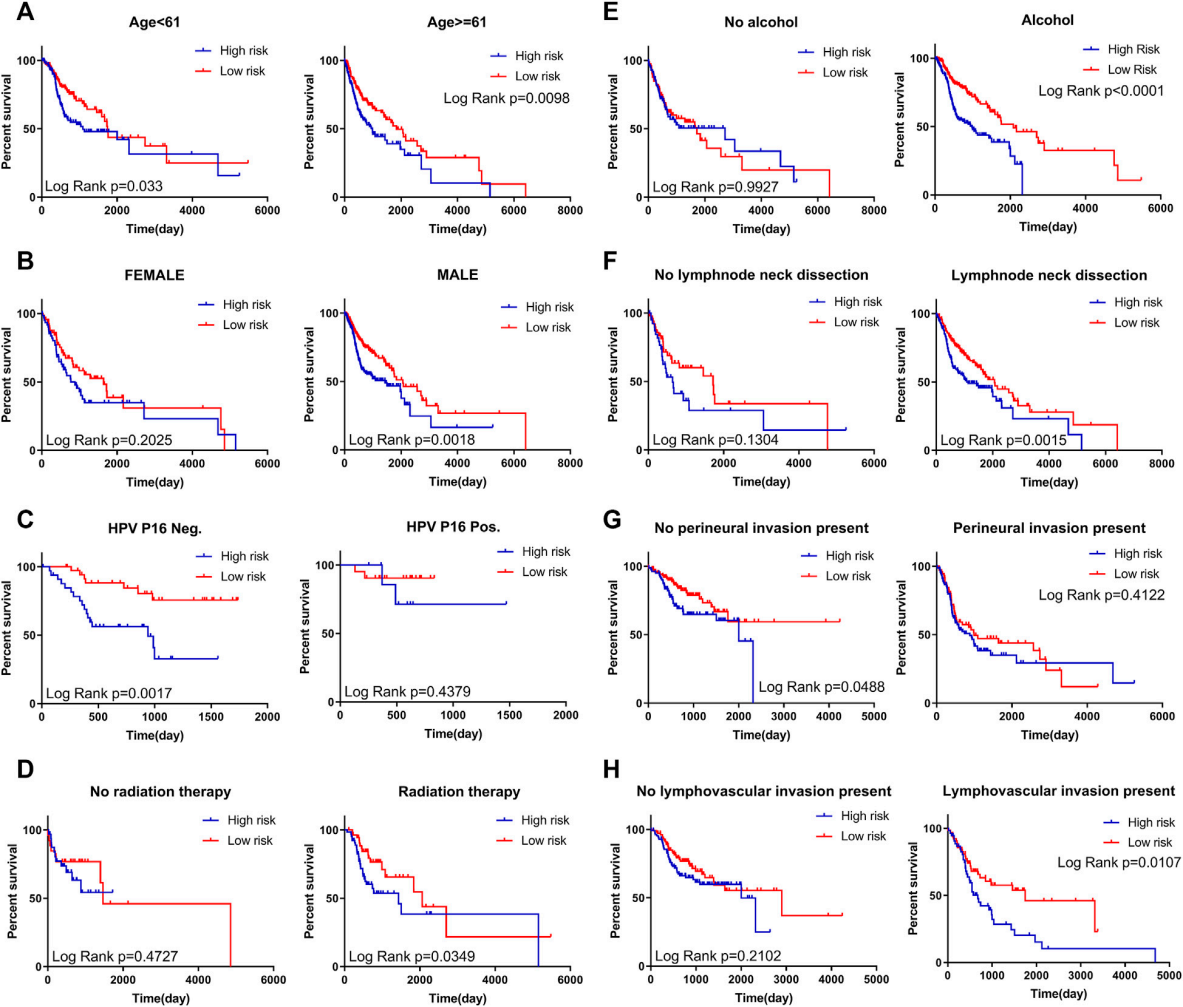
Kaplan–Meier curves for prognostic value of risk score signature for patients divided by each clinical feature. **(A)** Age **(B)** Gender **(C)** Expression status of HPV P16 **(D)** Status of radiation therapy **(E)** Alcohol status **(F)** Status of lymph node neck dissection **(G)** Presence of perineural invasion **(H)** Presence of lymph vascular invasion.

Stratified analysis for further data mining. As shown in Kaplan-Meier curve, regardless of age (<61 or ≥61), four-mRNA signature in HNSCC patients are stable prognostic markers, due to the poor prognosis of patients with a high-risk score (Figure 5A). Additionally, when patients were stratified into different subgroups based on gender and HPV P16 status the four-mRNA risk score remained an independent prognostic indicator for male HPV P16-negative (Figures 5B,C), suggesting that HNSCC may require further investigation. Similarly, when patients underwent radiation therapy during follow-up (with positive alcohol status and absence of perineural invasion), we could use risk scores to predict patient results, which showed that patients in the high-risk subgroups had poor survival (Figures 5D,E,G,H).

### Functional Enrichment Analysis of the Four-mRNA Signature in HNSCC

**FIGURE 6:**
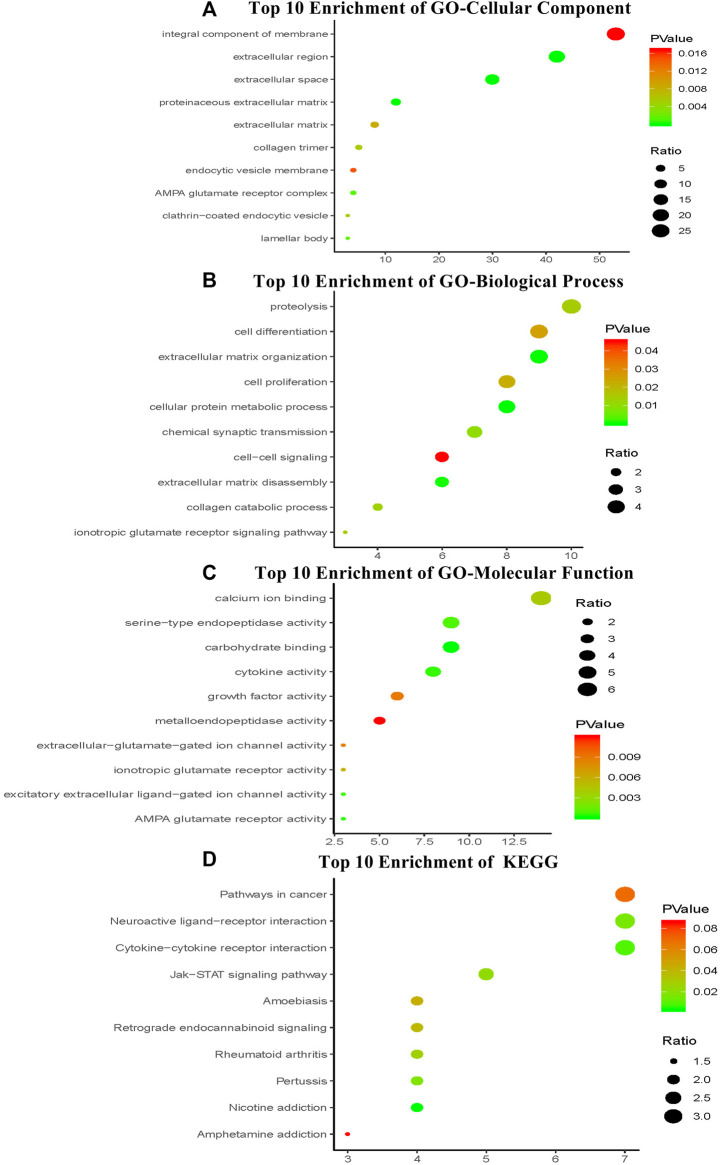
The top ten of GO term and pathway by target genes of four-mRNA signature in TCGA HNSCC cohort. The analysis was considered significant when P values were corrected for false discovery rate (FDR) **(A)** Parts of GO-CC categories of four-mRNA signature **(B)** Parts of GO-BP categories of four-mRNA signature **(C)** Parts of GO-MF categories of four-mRNA signature **(D)** Parts of KEGG pathway of four-mRNA signature.

We performed enrichment analysis to elucidate the biological function of the four-mRNA signature target genes. The GO categories comprised of three structured networks: biological processes (BP), cellular components (CC) and molecular function (MF). The top ten enriched functional analysis is shown in Figure 6. The top enriched biological process was proteolysis (associated with 10 genes); the top enriched cellular component and molecular function were integral component of membrane part (associated with 53 genes) and calcium ion binding (associated with 14 genes), respectively. Therefore, a total of 12 KEGG pathways were enriched by the four-mRNA signature. The significantly enriched KEGG pathways were the cancer-related pathways (associated with seven genes), cytokine-cytokine receptor interaction pathways (associated with seven genes) and neuroactive ligand-receptor interaction pathways (associated with seven genes).

## Discussion

Recent studies have shown that clinicopathological features such as gender, age, tumor margin status, and metastasis are not sufficient for accurately predict the prognosis of patients. As a result, more and more mRNAs might be examined as molecular markers at an increasing rate to predict cancer development and prognosis, indicating that its clinical significance needs to be explored [18]. For example, analysis of the prognostic value of a single lncRNA from qRT-PCR array of 84 gastric cancer patient samples found that higher level of BANCR could predict a poor prognosis for GC patients [19]. The high expression of FAM83H-AS1 is involved in the progression of bladder cancer and serves as a prognostic biomarker and potential therapeutic target for patients with bladder cancer [20]. Compared to single biomarker, integrating multiple biomarkers into the aggregation model could improve the prognostic value [21]. In particular, the expression of a single gene could be controlled by a variety of factors, and hence might not provide a strong predictive effect. Therefore, a statistical model was constructed gene signature contains multiple genes, combined with the effect of each component gene prediction, improve forecasting efficiency. This model has been used widely, and is superior to single biomarkers in predicting disease prognosis [22–24].

With the development of high-throughput genetic testing technology, we are entering a new era of big biological data [25]. Currently, RNA sequencing or micro array data for gene mutations and expression levels often use Cox proportional hazard regression models to construct new prognostic features [26, 27]. In this study, we attempted to apply GSEA using expression data of 57,072 genes of patients from TCGA-HNSCC cohort, and we found significant differences in five biological functions with *p*-values < 0.05. As mentioned above, we focused on EMT, using GSEA to select genes to predict patient survival rather than exploring a broad range of genes. Univariate and multivariate Cox regression analyses were performed to identify combinations of four genes with predictive values for HNSCC patients, rather than just one gene [21]. Compared to some known predictive biomarkers, the risk profile of this option may have a more targeted and prognostic ability to support positive clinical results and be an effective classification tool for HNSCC patients. In addition, this study used bioinformatics methods to explore the risk characteristics of mRNA and its clinical significance, providing a novel method for mining potential prognostic markers, which not only supports our previous understanding of HNSCC, but also lays the foundation for future studies. It should be noted that, Kaplan-Meier analysis showed that risk score on survival is small and appears even smaller than lymph vascular invasion or perineural invasion. Therefore, more comprehensive and homogeneous datasets are needed to assess the four-mRNA signature before clinical applications. Notably, the stratification analysis found that the four-mRNA signature could generate superior performances for predicting the survival benefit in patients with P16 negative or alcohol or age <61, indicating that the four-mRNA signature could be helpful in predicting HNSCC prognosis, especially in P16 negative or alcohol or age <61 patients. It is highly significant given that P16 positive tumors typically have a good prognosis regardless of stage or high-risk pathologic features, this prognostic mRNA signature classifier for P16 negative-related HNSCC may help clinicians to pinpoint those HNSCC patients at high risk of unfavorable OS. EMT is recognized to be important in cancer cell migration/metastasis. For example, to metastasize, disseminated mesenchyme-like tumor cells of well-differentiated carcinomas must regain their epithelial function (invasion and metastasis) [28, 29]. The disruption of epithelial-cell homeostasis leading to aggressive cancer progression is correlated with the loss of epithelial characteristics and the acquisition of a migratory phenotype, known to EMT, and is considered to be a crucial event in malignancy [30, 31]. EMT and mesenchymal-related gene expression are associated with aggressive breast cancer subtypes and poor clinical outcome in breast cancer patients [32, 33]. Recently, some studies have reported the occurrence and development of gene expression induced EMT and metastasis in HNSCC [34, 35]. Additionally, several studies have been conducted which have predicted that in HNSCC patients, survival is associated with EMT. For instance, TEAD4 might act as a putative oncogenic gene by enhancing the proliferation, migration, and invasion of HNSCC cancer cells [36]. However, the EMT gene signature used to predict HNSCC prognosis have not been established. In the present study, we reported an EMT gene signature of four genes (*PPIB, BASP1, WIPF1 and PLOD2*), identified using bioinformatics methods which demonstrate the prognostic value of HNSCC. Of the four candidate genes, three have positively correlated coefficients in the prognostic model and correlated with poor survival. PPIB, called cyclophilin B, regulates the protein conformation of its substrate through prolyl cis–trans-isomerization in endoplasmic reticulum (ER) lumen and nucleus, and possesses multiple functions, including chemotaxis and prolactin signaling [37–39]. Additionally, it is a novel wild-type p53 (p53WT)-inducible gene [40]. In cancer biology, PPIB is associated with malignant progression and regulation of genes involved in the pathogenesis of gastric cancer [41],hepatocellular carcinoma [42], pancreatic cancer [43] and is considered as a candidate biomarker for these cancers. However, the exact molecular mechanism by which PPIB leads to cancer cell survival is unclear. BASP1 (brain acid soluble protein 1), was initially isolated from 23,000 brain extracts. BASP1 comprises an effector domain which dynamically couples to the plasma membrane and is also involved in neuronal sprouting process [44]. Recently, some reports have identified BASP1 as a transcriptional co-suppressor for WT1 protein [45]. WT1 can promote tumor cell proliferation, inhibition of apoptosis, and interacts with cytoskeletal proteins to promote migration, invasion and angiogenesis [46]. *WT1* gene is expressed highly abnormally in a variety of tumors, including breast [47], thyroid [48, 49], non-small cell lung cancers [50], and HNSCC [51], and is considered to have the characteristics of an oncogene. PLOD2, also called LH2, is the key enzyme mediating the formation of stabilized collagen cross-links [52]. Gain and loss of function studies show that LH2 hydroxylated telopeptide lysine residues on collagen, shifted the tumor stroma toward higher levels of hydroxylysine aldehyde–derived collagen cross-links (HLCCs), lower levels of lysine aldehyde–derived cross-links (LCCs), increased tumor stiffness, and enhanced tumor cell invasion and metastasis [53, 54]. PLOD2 is overexpressed in different cancers such as bladder cancer [55], renal cell carcinoma [56], and oral carcinoma [57], and is closely associated with poor prognosis [58]. All these three genes are associated with EMT in cancer. In contrast, WIPF1 confers an onco-protective effect. WIPF1, also known as WIP, the WASP-interacting protein (WIP) drives the oncogenic activity of mutant p53. WIPF1 knockdown in glioblastoma and breast cancer cells expressing mtp53 greatly reduced the proliferation and growth capacity of cancer stem cell-like cells and reduced the expression of cancer stem cell-like markers (CD44, CD133 or TAZ/YAP). WIPF1 knockdown inhibits the growth of glioblastoma tumor cells and breast cancer cells *in vivo* [59]. Since EMT is affected in HNSCC, the targeted genes might be useful in controlling cancer. Finally, the prognostic value of the four genes in HNSCC still needs further experimental studies to elucidate biological function.

## Conclusion

In conclusion, we identified and verified a four-gene risk signature related to EMT that can predict the survival of HNSCC patients, wherein a high-risk score shows poor patient prognosis, suggesting that this gene signature could be a potential new biomarker for HNSCC prognosis. Further investigation of these genes will provide theoretical guidance for basic research and for the clinical treatment of HNSCC.

## Data Availability

The original contributions presented in the study are included in the article/Supplementary Material, further inquiries can be directed to the corresponding authors.
